# Conjunctival Lymphangioma: A Case Report and Brief Review of the Literature

**DOI:** 10.1155/2012/836573

**Published:** 2012-05-17

**Authors:** Mariana Seca, Pedro Borges, Pedro Reimão, Miguel Gomes, Angelina Meireles

**Affiliations:** Departmento de Oftalmologia, Hospital de Santo António, Centro Hospitalar do Porto, Largo Professor Abel Salazar, Edifício Neoclássico, 4099-001 Porto, Portugal

## Abstract

*Background*. Lymphangioma is a rare venolymphatic lesion, characterized by dilation of lymphatic vessels. It may occur as an isolated lesion or, more often, represent the surface component of a deep orbital lymphangioma. *Case*. We report a case of a conjunctival lymphangioma on a 58-year-old male that had simultaneously an upper respiratory tract infection. Excision and biopsy confirmed the nature of the lesion, and there has been no relapse to date. *Conclusion*. Conjunctival lymphangioma is a rare condition in which the diagnose, must be kept in mind in patients with a red eye resistance to topical therapy and in association with an upper respiratory tract infection. Finally, it is also necessary to be aware of possible recurrence of the lesion.

## 1. Introduction

Lymphangioma is an uncommon venolymphatic lesion, also defined as vascular hamartoma of lymphatic origin [1, 2]. The term lymphangioma is appropriately used when there is hemodynamic isolation, and so the injury is not related to arterial or venous system [1]. It is characterized by dilation of lymphatic vessels that contain lymph, and it appears as a multiloculated cyst-like lesion.

Etiology of lymphangioma of the conjunctiva is not yet clear. It is usually an unilateral sporadic occurrence unless associated with the Turner syndrome or the Nonne-Milroy-Meige disease [3]. Most frequently seen in the bulbar conjunctiva, it may occur as either a solitary or a multifocal conjunctival lesions. In most cases, it represents the surface component of a deeper diffuse orbital lymphangioma [4].

The lymphangioma can be exacerbated by intralesion hemorrhage resulting in large “chocolate cysts” or by episodes of upper airway infections, resulting in lymphoid hyperplasia [1, 5].

This paper describes a clinical case of a patient with an isolated conjunctival lymphangioma and its ophthalmological implications.

## 2. Case Report

A 55-year-old man presented a lush conjunctival hyperaemia and chemosis of the inferior bulbar conjunctiva in the left eye accompanied with dry eye symptoms, on March 2011. This lesion has been described for the first time three months earlier, and it had been the reason for five emergency hospital visits. In this period the patient has been treated with different drops and ointments, including topical steroids, anti-inflammatory, antibiotics, and eye lubricants, but none has proven any efficacy.

**Figure 1:**
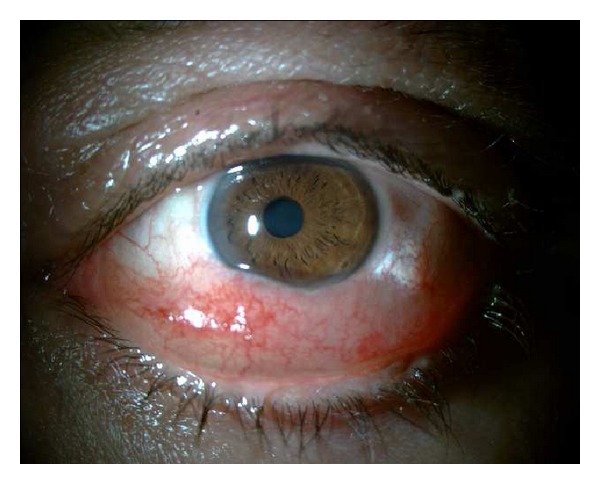
Preoperative biomicroscopy.

**Figure 2:**
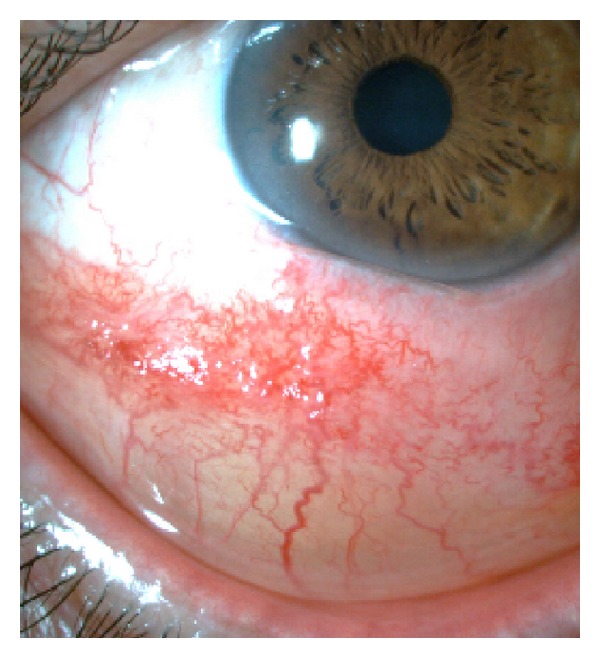
Preoperative biomicroscopy.

On March 2011, examination of the left eye disclosed an unilateral swollen and hyperemic inferior bulbar conjunctiva extending to both canthi; this was a light-pink-coloured lesion, painless, compressible and with an underlying transparent content (Figures 1 and 2); the inferior border of the mass was not identified. 

In the patient's medical history is important to highlight a kidney transplant in 1999 by the Alport syndrome that is currently being treated with corticosteroids, cyclosporine, and azathioprine. 

On ophthalmic examination, the best corrected visual acuity was 20/25 in both eyes. The biomicroscopy of the left eye revealed a punctate keratitis and the fundoscopy was normal. Intraocular pressure was 15 mmHg and pupils' reflexes were normal. The patient had no proptosis and no limitation on the eye movements. No signs of eye alterations associated with Alport's disease were found. No other systemic symptoms were pointed out by the patient.

**Figure 3:**
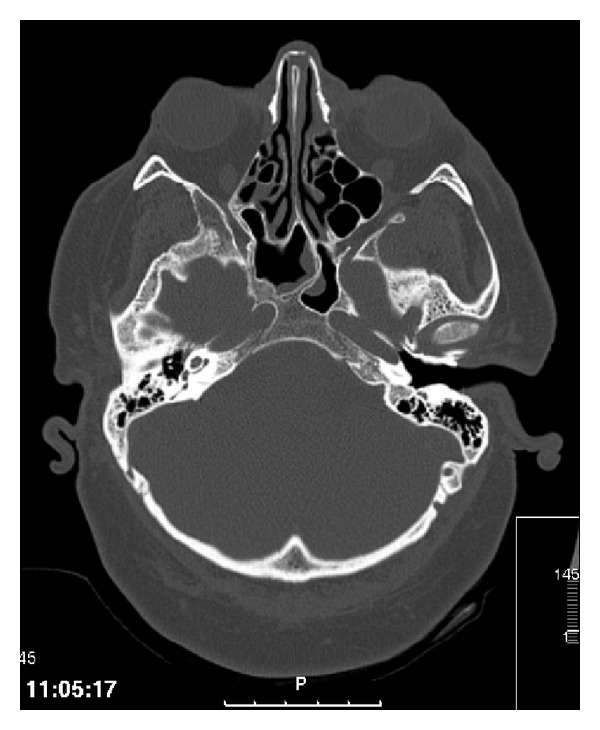
Computed tomography scan of the orbits.

**Figure 4:**
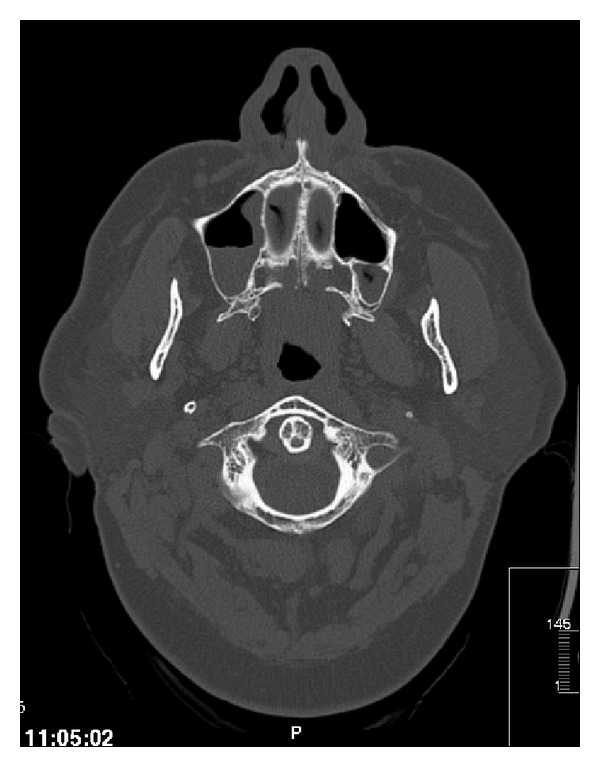
Computed tomography scan of the orbits.

A computed tomography scan of the orbits and brain described a right and left maxillary and sphenoid sinuses, suggestive of sinus inflammation, with no involvement of the orbit (Figures 3 and 4).

**Figure 5:**
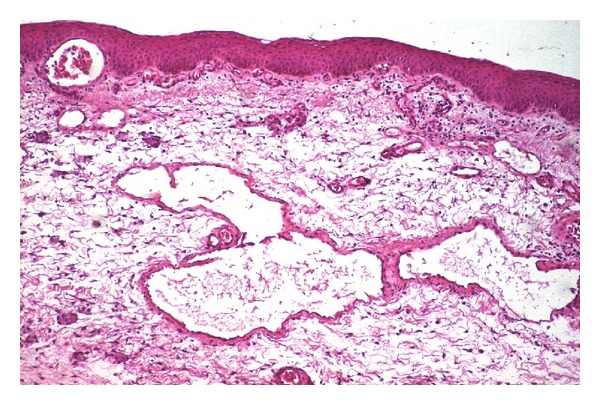
Histopathological finding of the inferior bulbar conjunctiva (hematoxylin and eosin stain, ×100).

On May 2011, an incisional biopsy was performed under local anaesthesia which proved to be diagnostic and therapeutic. Histology showed a lymphatic proliferation and ectasia with a network of empty bloodless channels lined by flattened endothelium with the presence of some blood vessels and inflammatory infiltrate (haematoxylin and eosin stain, ×100; Figure 5), which is consistent with the diagnosis of conjunctival lymphangioma.

**Figure 6:**
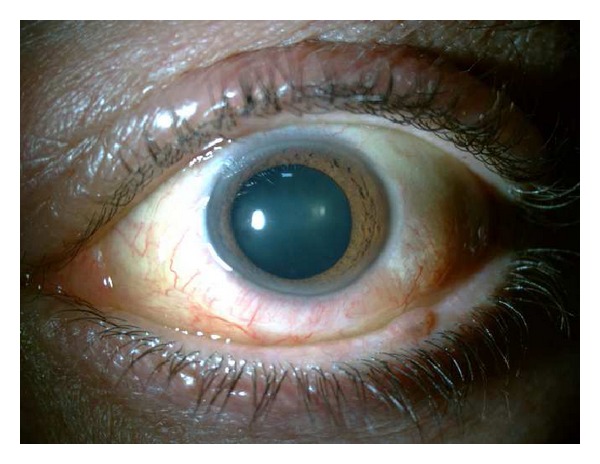
Postoperative biomicroscopy.

**Figure 7:**
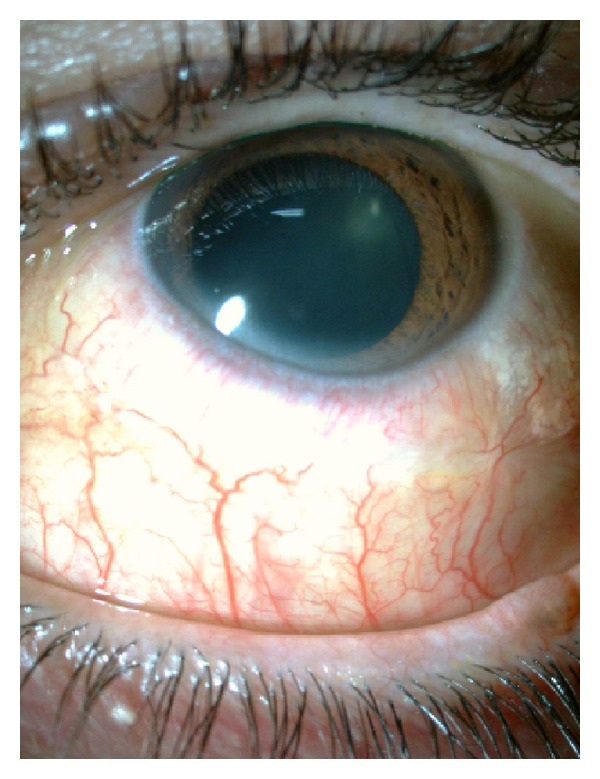
Postoperative biomicroscopy.

The excision of the lesion led to the resolution of the case (Figures 6 and 7). Meanwhile the upper respiratory tract infection, which is likely to have exacerbated the size of the lymphangioma was resolved.

Ten months later the patient remains asymptomatic with no signs of recurrence of the lesion.

## 3. Discussion

Lymphangiomas are benign, hamartomatous, vascular tumours that may affect the orbits, eyelids, or conjunctiva [6–9]. They represent 1–3% of orbital masses [7, 8]. Coexisting lymphangiomas of the face, nasal sinuses, nasal cavity, or palate may suggest the diagnosis [7].

Isolated conjunctival lymphangioma is a rare condition with an average age of presentation at 25 years old (range from birth to 65 years) [9]. Rootman et al. [8] presented only a single case of a lymphangioma isolated to the lower fornix, in a series of 13 cases of orbital-adnexal lymphangiomas.

Orbital lymphangiomas often present with proptosis, ptosis, and impaired extraocular movements. Conjunctival lymphangiomas generally appear as a visible mass without affecting vision, or the globe [10], as occurred with our patient.

Lymphangiectasia can lead to irritation and redness from desiccation of the overlying conjunctival epithelium, epiphora, if the lacrimal puncta become functionally occluded by overhanging conjunctiva, blurred vision, and pain [11].

As described by Duke-Elder [12], conjunctival lymphangiectasia has two principal manifestations: a cystic lesion of the conjunctiva, which may mimic allergic chemosis, and a beaded dilation of lymphatic vessels with a string of pearl appearance. Unless the latter type acquires communication with a conjunctival vein and becomes hemorrhagic, simple dilations of the conjunctival lymphatic vessels are asymptomatic and usually unnoticed.

In a review of 13 cases of orbital-adnexal lymphangiomas, Rootman et al. [8] classified these lesions into superficial (as in our case), deep, and combined. Superficial lesions consisted of isolated multicystic vascular abnormalities of cosmetic significance only. The deep and combined lesions were found to be more symptomatic.

Biopsy with a histopathological report has an essential role in the diagnosis. Histopathologically, lymphangioma is a nonencapsulated, irregular mass, lined by endothelial cells, composed of numerous cyst-like channels that contain clear fluid, blood, or a combination of both. Separating the channels is loose connective tissue that contains aggregates of small lymphocytes [9]. In our case the biopsy was the diagnostic key.

Treatment of superficial lymphangioma by surgical excision is often curative [11, 13, 14] as occurred with our patient. The larger classical lesions tend to recur following surgery, as the extent of subcutaneous involvement may be difficult to define, making complete excision impossible [15].

Alternative treatments for conjunctival lymphangiectasia have been described. In a case report by Jordan and coworkers [4], a carbon dioxide laser was used to successfully treat what was described as a conjunctival lymphangioma. Behrendt and colleagues [16] used beta irradiation to effectively treat a case of conjunctival lymphangioma. A case report from Egypt, by Wasfy [17], describes the use of cryotherapy to successfully treat a case of conjunctival lymphangiectasia. Liquid nitrogen cryotherapy has been proven safe and effective for conjunctival lymphangiectasia [18]. Conservative, nonsurgical management with eventual spontaneous resolution has also been reported [19].

We did not find any correlation between Alport's syndrome and conjunctival lymphangioma in the literature [20, 21].

This paper presents a rare case of a lymphangioma isolated to the bulbar conjunctiva that was a differential diagnosis of red eye resistant to therapy. The upper respiratory infection may have exacerbated the signs of the conjunctival lesion. These lesions are a diagnostic challenge, and the biopsy has an essential role in the accurate diagnosis. Surgical excision is one of the treatment options, and it was effective in our patient. It ishowever necessary to be aware of possible recurrence of the lesion.
